# Apgar Score and Neurodevelopmental Outcomes at Age 5 Years in Infants Born Extremely Preterm

**DOI:** 10.1001/jamanetworkopen.2023.32413

**Published:** 2023-09-06

**Authors:** Harald Ehrhardt, Adrien M. Aubert, Ulrika Ådén, Elizabeth S. Draper, Anna Gudmundsdottir, Heili Varendi, Tom Weber, Michael Zemlin, Rolf F. Maier, Jennifer Zeitlin

**Affiliations:** 1 Division of Neonatology and Pediatric Intensive Care Medicine, Department of Pediatrics and Adolescent Medicine, University Medical Center Ulm, Ulm, Germany; 2 Université Paris Cité, Inserm, National Research Institute for Agriculture, Food and the Environment, Centre for Research in Epidemiology and Statistics, Obstetrical Perinatal and Pediatric Epidemiology Research Team, Paris, France; 3 Department of Women’s and Children’s Health, Karolinska Institutet, Stockholm, Sweden; 4 Department of Health Sciences, University of Leicester, Leicester, United Kingdom; 5 Department of Women’s and Children’s Health, Karolinska Institutet, Stockholm, Sweden; 6 University of Tartu, Tartu University Hospital, Tartu, Estonia; 7 University of Copenhagen, Copenhagen, Denmark; 8 Saarland University Medical Center, Hospital for General Pediatrics and Neonatology, Homburg, Germany; 9 Children’s Hospital, University Hospital, Philipps University Marburg, Marburg, Germany

## Abstract

**Importance:**

The Apgar score is used worldwide as an assessment tool to estimate the vitality of newborns in their first minutes of life. Its applicability to estimate neurodevelopmental outcomes in infants born extremely preterm (EPT; <28 weeks’ gestation) is not well established.

**Objective:**

To investigate the association between the Apgar score and neurodevelopmental outcomes in infants born EPT.

**Design, Setting, and Participants:**

This cohort study was conducted using data from the Effective Perinatal Intensive Care in Europe-Screening to Improve Health in Very Preterm Infants in Europe (EPICE-SHIPS) study, a population-based cohort in 19 regions of 11 European countries in 2011 to 2012. Clinical assessments of cognition and motor function at age 5 years were performed in infants born EPT and analyzed in January to July 2023.

**Exposures:**

Apgar score at 5 minutes of life categorized into 4 groups (0-3, 4-6, 7-8, and 9-10 points).

**Main Outcomes and Measures:**

Cognitive and motor outcomes were assessed using the Wechsler Preschool and Primary Scale of Intelligence test of IQ derived from locally normed versions by country and the Movement Assessment Battery for Children–Second Edition. Parents additionally provided information on communication and problem-solving skills using the Ages and Stages Questionnaire, third edition (ASQ-3). All outcomes were measured as continuous variables.

**Results:**

From the total cohort of 4395 infants born EPT, 2522 infants were live born, 1654 infants survived to age 5 years, and 996 infants (478 females [48.0%]) followed up had at least 1 of 3 outcome measures. After adjusting for sociodemographic variables, perinatal factors, and severe neonatal morbidities, there was no association of Apgar score with IQ, even for scores of 3 or less (β = −3.3; 95% CI, −10.5 to 3.8) compared with the score 9 to 10 category. Similarly, no association was found for ASQ-3 (β = −2.1; 95% CI, −24.6 to 20.4). Congruent results for Apgar scores of 3 or less were obtained for motor function scores for all children (β = −4.0; 95% CI, −20.1 to 12.1) and excluding children with a diagnosis of cerebral palsy (β = 0.8, 95% CI −11.7 to 13.3).

**Conclusions and Relevance:**

This study found that low Apgar scores were not associated with longer-term outcomes in infants born EPT. This finding may be associated with high interobserver variability in Apgar scoring, reduced vitality signs and poorer responses to resuscitation after birth among infants born EPT, and the association of more deleterious exposures in the neonatal intensive care unit or of socioeconomic factors with greater changes in outcomes during the first 5 years of life.

## Key Points

**Question:**

Is the Apgar score at 5 minutes of life associated with neurodevelopmental outcomes in infants born extremely preterm?

**Findings:**

In this cohort study including 996 infants at less than 28 weeks’ gestation followed-up at 5 years, low Apgar scores were not associated with cognitive or motor outcomes.

**Meaning:**

These findings suggest that the Apgar score may not have good prognostic value for long-term outcomes in infants born extremely preterm and should be interpreted with caution as a basis for treatment decisions.

## Teaser

This cohort study investigates the association of Apgar score at age 5 minutes with neurodevelopmental outcomes among infants born extremely preterm.

## Introduction

The Apgar score was originally developed by Virginia Apgar in 1953 for the standardized evaluation of the newborn infant’s clinical condition at 1 minute after birth and the need for resuscitation measures.^1^ Although it was not the developer’s intention to predict neurodevelopmental outcomes, Apgar scores at 5 and 10 minutes of life were later found to be associated with mortality and acute morbidities in infants born at term.^2,3,4^ After the Apgar score’s original description, advances in medical care have dramatically improved neonatal outcomes. Nonetheless, the Apgar score remains the most commonly used assessment tool, and low Apgar scores at 5 minutes after birth are uniformly associated with an increased risk of mortality, acute morbidities, impaired neurodevelopmental outcomes, and educational support services in infants born at term.^2,3,4,5,6,7,8,9,10,11^

A similar association of low 5-minute Apgar scores after birth and increased risks of mortality and cerebral palsy (CP) was observed in infants born preterm, although the strength of the association decreased at lower gestational ages.^5,12,13,14^ A 2022 study^12^ by a multinational collaboration did not reveal an association between low Apgar scores and severe neurologic injury, defined as grade 3 or 4 intraventricular hemorrhage (IVH) or cystic periventricular leukomalacia (CPVL), in infants born extremely preterm (EPT; ie, those born at less than 28 weeks’ gestation). These results raise questions about the association between the Apgar score and neurodevelopmental outcomes in this high-risk population given that these brain lesions constitute the main risk factors associated with neurodevelopmental impairment.^15,16,17,18^

Overall, the prognostic accuracy of the Apgar score for longer-term outcomes in infants born EPT has not been conclusively studied to our knowledge. The objective of this study was to investigate the association between low Apgar scores at 5 minutes of life and cognitive and motor outcomes at age 5 years among children born EPT within the population-based Effective Perinatal Intensive Care in Europe-Screening to Improve Health in Very Preterm Infants in Europe (EPICE-SHIPS) cohort.

## Methods

### Study Design and Ethics Approval

This cohort study used data from the prospective population-based EPICE study, which enrolled all stillbirths and live births from 22 + 0 to 31 + 6 weeks’ gestation across 19 regions of 11 European countries for a 12-month period between April 2011 and September 2012 with the exception of the French region, where data collection occurred over 6 months.^19^ Ethics approval was obtained from regional or hospital ethics committees as required by national legislation. The European study was approved by the French Advisory Committee on Use of Health Data in Medical Research and the French National Commission for Data Protection and Liberties. This ethical approval covers analyses of perinatal determinants of neurodevelopment at 5 years, including this study. Parental informed consent was obtained for the SHIPS study on follow-up at age 5 years, which includes analyses of sociodemographic and perinatal determinants of child development and well-being. This study adheres to the Strengthening the Reporting of Observational Studies in Epidemiology (STROBE) reporting guideline for observational studies.

Obstetric and neonatal baseline and outcome data up to discharge home from the neonatal unit were retrieved from patient records using a standardized questionnaire with pretested definitions. At age 5 years, children born at less than 28 weeks’ gestation were offered a standardized clinical assessment evaluating neurocognitive and motor functioning using the Wechsler Preschool and Primary Scale of Intelligence–Revised, Third, or Fourth Edition depending on locally normed tests and the Movement Assessment Battery for Children–Second Edition (MABC-2). Assessments were executed by local routine follow-up programs or the SHIPS research team depending on the organization of follow-up in each country.^20^ For all children in the cohort, caregivers filled in a questionnaire that included 2 domains (the communication and problem-solving domains) of the Ages and Stages Questionnaire, third edition (ASQ-3). Further information on family characteristics and child health status was obtained by this parental questionnaire.

### Study Population

The population for this analysis included all children born EPT in the cohort with follow-up at age 5 years born between 22 and 27 weeks’ gestation. The EPT subgroup was selected because of the availability of standardized clinical assessments of cognitive and motor function.

### Exposure

Our exposure was the Apgar score at 5 minutes of life. We classified Apgar scores into 4 categories based on commonly used cutoffs (0-3, 4-6, 7-8, and 9-10 points) to enable comparison with other studies.^5,10,13,14,21^

### Definition of Outcomes and Covariates

Our primary outcomes were full-scale IQ scores calculated using local norms. For the MABC-2, we used United Kingdom norms^22^ given that not all countries had local norms. We used the total component score, which is the sum of component scores of standardized items in each domain of the test (manual dexterity, aiming and catching, and balance); the score had a range from 14 to 108. We also used information on whether the child had a diagnosis of CP as reported by caregivers in the parental questionnaire, except in France, where this information came from a clinical assessment of CP by the study team. Training of assessors and strategies for reporting consistent results and managing missing or incomplete data were reported previously.^20,23^ Additionally, 2 domains of the ASQ-3, with questions selected from 60- and 72-month tests, were used to assess communication and problem-solving skills. These 2 domains included 9 questions. For each question on the child’s abilities, responses are not yet, sometimes, and yes, scored 0, 5, or 10 points, respectively, for a total range of 0 to 180.

Maternal and socioeconomic characteristics included maternal age and parity at childbirth, maternal education, maternal country of birth, parental cohabiting status, and employment status of parents. Gestational age was based on the best obstetrical estimate; when there were several estimates, we used the following hierarchy: in vitro fertilization treatment, ultrasound based on earliest estimate, last menstrual period, fundal height measurement, and neonatal assessment at birth. Pregnancy and neonatal variables included single or multiple pregnancy; sex; small for gestational age status, classified as birthweight less than the third, third to ninth, and tenth or greater percentile of intrauterine references developed for the cohort^24^; premature rupture of membranes more than 12 hours before the onset of labor; and antenatal steroid administration (at least 1 dose before delivery irrespective of the time interval to delivery). The following severe neonatal morbidities were considered in analyses as done previously in this cohort: IVH grade III and IV, CPVL, or both; retinopathy of prematurity of stage 3 or greater; necrotizing enterocolitis requiring surgical therapy or peritoneal drainage; and moderate and severe bronchopulmonary dysplasia with ongoing need for respiratory support, supplemental oxygen, or both at 36 weeks.^25,26^

### Statistical Analysis

We compared the cohort’s baseline sociodemographic and clinical characteristics between Apgar score categories. *P* values to assess differences between groups were based on ordered logistic models to control for country of birth given that there were differences in the distribution of Apgar score categories by country. Associations between the Apgar score in 4 categories and IQ and MABC-2 standardized score modeled as continuous variables were investigated with linear regression. We constructed 4 models: (1) no adjustments except for country as a fixed effect (termed unadjusted), (2) adjustments for country and additionally on sociodemographic factors, (3) adjustments for country and sociodemographic factors from models 1 and 2 plus adjustment on perinatal factors, and (4) a full model with adjustments for country, sociodemographic factors, perinatal factors, and additionally on severe neonatal morbidities to take into consideration postnatal factors.^23,26,27,28,29^ To avoid collinearity in adjusted models, we included maternal age, education, and country of birth as sociodemographic factors; gestational age, SGA, child sex, and multiplicity as perinatal factors; and a composite of severe morbidity and BPD as neonatal factors. We applied inverse probability weighting (IPW) to account for loss to follow-up as done previously on this population.^23,30,31^ Missing data for most covariates was less than 4% (eg, mother’s age: 4 of 996 individuals [0.4%] or any antenatal steroids: 7 individuals [0.7%]); those with missing data greater than 4% included maternal education (40 individuals [4.0%]), parents’ cohabiting (59 individuals [5.8%]) and employment (66 individuals [6.6%]) status, and Apgar score (78 individuals [7.8%]). We used multiple imputation by chained equations for weights (20 imputed data sets) and for main models (20 imputed data sets).^32^ We did not impute missing data on IQ or MABC-2 scores. In sensitivity analyses, we ran models without imputation and IPW. Additionally, we redid analyses without multiple imputation for missing Apgar scores and after excluding Belgium and Sweden, which had different patterns of Apgar scores compared with other countries. All analyses were done with the statistical software Stata version 15.0 (StataCorp). *P* < .05 was considered statistically significant using 2-sided tests. Data were analyzed from January to July 2023.

## Results

### Study Population and Apgar Score Distribution

**Figure 1.**
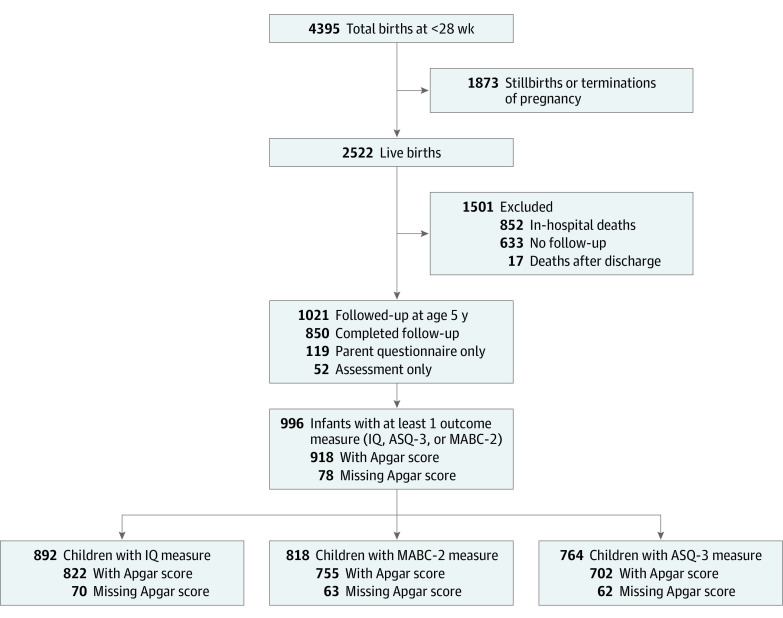
Study Flowchart ASQ-3 indicates Ages and Stages Questionnaire, third edition; MABC-2, Movement Assessment Battery for Children–Second Edition.

Of 4395 total EPT births in the cohort, 2522 infants were live born; 851 deaths occurred during the neonatal hospitalization, and 17 deaths occurred before age 5 years. A total of 1654 children survived to 5 years, and 1021 individuals were followed up (Figure 1); 996 children (478 females [48.0%]; 318 children born at less than 26 weeks [31.8%]; 154 children SGA <third percentile [15.5%]) had at least 1 of the follow-up instruments available (IQ score, ASQ-3 score from the parental questionnaire, or the MABC-2 assessment) (eTable 1 in Supplement 1). Of these individuals, 918 children had data on the Apgar score at 5 minutes of life (Figure 1). Data on IQ were available for 892 infants, on MABC-2 for 818 infants, and on ASQ-3 for 764 infants (Figure 1). Most infants had an Apgar score of 9 to 10 (306 individuals [33.3%]) or 7 to 8 (383 individuals [41.7%]), whereas 180 infants (19.6%) had scores of 4 to 6 and 49 infants (5.3%) had scores of 0 to 3 (Table 1). The follow-up rate varied by region, from 138 of 359 infants (38.4%) at the UK to 38 of 40 infants (95.0%) at Estonia, yielding an overall follow-up rate of 1021 of 1654 eligible infants (61.7%) at age 5 years.

**Table 1.** Patient Characteristics by Apgar Score

Characteristic	Patients^a^					*P* value^c^
	Total, No. (N = 918)	Apgar score, No. (row %)^b^				
		0-3 (n = 49 [5.3%])	4-6 (n = 180 [19.6%])	7-8 (n = 383 [41.7%])	9-10 (n = 306 [33.3%])	
Maternal age, y						
<25	106	10 (9.4)	16 (15.1)	47 (44.3)	33 (31.1)	.71
25-34	550	20 (3.6)	116 (21.1)	222 (40.4)	192 (34.9)	
≥35	259	17 (6.6)	48 (18.5)	113 (43.6)	81 (31.3)	
Maternal living situation						
Single or other	126	6 (4.8)	27 (21.4)	47 (37.3)	46 (36.5)	.88
Married or cohabitation	738	42 (5.7)	142 (19.2)	313 (42.4)	241 (32.7)	
Maternal education, ISCED score						
Low (0-2)	159	7 (4.4)	31 (19.5)	55 (34.6)	66 (41.5)	.17
Intermediate (3-5)	380	26 (6.8)	76 (20.0)	163 (42.9)	115 (30.3)	
High (6-8)	340	12 (3.5)	67 (19.7)	147 (43.2)	114 (33.5)	
Parents employed						
Yes, both	742	32 (4.3)	143 (19.3)	316 (42.6)	251 (33.8)	.03
No, 1 or both	114	14 (12.3)	25 (21.9)	41 (36.0)	34 (29.8)	
Maternal country of birth						
Same country	707	30 (4.2)	140 (19.8)	298 (42.1)	239 (33.8)	.05
Other European country	55	3 (5.5)	10 (18.2)	21 (38.2)	21 (38.2)	
Non-European country	154	15 (9.7)	30 (19.5)	63 (40.9)	46 (29.9)	
Parity						
Primiparous	536	24 (4.5)	101 (18.8)	214 (39.9)	197 (36.8)	.01
Multiparous	371	25 (6.7)	78 (21.0)	166 (44.7)	102 (27.5)	
Type of pregnancy						
Singleton	662	38 (5.7)	145 (21.9)	267 (40.3)	212 (32.0)	.21
Multiple	256	11 (4.3)	35 (13.7)	116 (45.3)	94 (36.7)	
Gestational age, completed wk						
22-24^d^	119	12 (10.1)	30 (25.2)	52 (43.7)	25 (21.0)	<.001
25	163	11 (6.7)	47 (28.8)	65 (39.9)	40 (24.5)	
26	260	10 (3.8)	39 (15.0)	118 (45.4)	93 (35.8)	
27	376	16 (4.3)	64 (17.0)	148 (39.4)	148 (39.4)	
SGA, percentile						
<3	145	6 (4.1)	20 (13.8)	59 (40.7)	60 (41.4)	.02
3-9	72	7 (9.7)	14 (19.4)	29 (40.3)	22 (30.6)	
≥10	701	36 (5.1)	146 (20.8)	295 (42.1)	224 (32.0)	
Sex						
Male	479	26 (5.4)	109 (22.8)	196 (40.9)	148 (30.9)	.006
Female	439	23 (5.2)	71 (16.2)	187 (42.6)	158 (36.0)	
PPROM						
No	672	34 (5.1)	132 (19.6)	272 (40.5)	234 (34.8)	.29
Yes	235	15 (6.4)	45 (19.1)	105 (44.7)	70 (29.8)	
ANS						
No	101	9 (8.9)	23 (22.8)	52 (51.5)	17 (16.8)	<.001
Yes	811	40 (4.9)	156 (19.2)	329 (40.6)	286 (35.3)	
Any congenital anomaly						
No	837	46 (5.5)	160 (19.1)	350 (41.8)	281 (33.6)	.53
Yes	81	3 (3.7)	20 (24.7)	33 (40.7)	25 (30.9)	
Country						
Belgium	67	7 (10.4)	9 (13.4)	29 (43.3)	22 (32.8)	<.001
Denmark	52	1 (1.9)	5 (9.6)	6 (11.5)	40 (76.9)	
Estonia	37	1 (2.7)	16 (43.2)	19 (51.4)	1 (2.7)	
France	154	10 (6.5)	26 (16.9)	46 (29.9)	72 (46.8)	
Germany	71	1 (1.4)	21 (29.6)	29 (40.8)	20 (28.2)	
Italy	131	4 (3.1)	25 (19.1)	76 (58.0)	26 (19.8)	
Netherlands	75	4 (5.3)	16 (21.3)	40 (53.3)	15 (20.0)	
Poland	50	3 (6.0)	17 (34.0)	21 (42.0)	9 (18.0)	
Portugal	111	3 (2.7)	14 (12.6)	49 (44.1)	45 (40.5)	
United Kingdom	129	4 (3.1)	23 (17.8)	59 (45.7)	43 (33.3)	
Sweden	41	11 (26.8)	8 (19.5)	9 (22.0)	13 (31.7)	

Abbreviations: ANS, antenatal steroids; ISCED, International Standard Classification of Education; PPROM, preterm premature rupture of membranes; SGA, small for gestational age.

^a^ The total number of patients with Apgar score will differ given missing values for individual covariables.

^b^ Data are given as percentages of rows separated by Apgar score.

^c^ *P* values were computed after adjusting for country using ordered logistical regression models.

^d^ Includes 14 children born at 23 weeks of gestational age.

Lower Apgar score categories were more frequent with parental unemployment, foreign maternal country of birth, multiparity, lower gestational age, small for gestational age, male sex, no receipt of antenatal steroids, and country of birth (Table 1). Additionally, BPD and ROP were more frequently observed among children with lower Apgar scores; for example, among 296 infants with BPD, 20 infants (6.8%) had Apgar scores less than 4 and 73 infants (24.7%) had Apgar scores of 4 to 6, while among 602 infants without BPD, 29 infants (4.8%) had Apgar scores less than 4 and 103 infants (17.1%) had Apgar scores of 4 to 6. For other severe neonatal morbidities of IVH or CPVL and surgical necrotizing enterocolitis, no differences were detected (Table 2).

**Table 2.** Neonatal Outcomes by Apgar Score

Characteristic	Patients^a^					*P* value^c^
	Total, No.	Apgar score, No. (row %)^b^				
		0-3 (n = 49 [5.3%])	4-6 (n = 180 [19.6%])	7-8 (n = 383 [41.7%])	9-10 (n = 306 [33.3%])	
IVH grade III-IV or CPVL						
No	790	40 (5.1)	150 (19.0)	331 (41.9)	269 (34.1)	.08
Yes	121	9 (7.4)	29 (24.0)	49 (40.5)	34 (28.1)	
ROP ≥stage 3						
No	800	37 (4.6)	151 (18.9)	335 (41.9)	277 (34.6)	.007
Yes	107	12 (11.2)	27 (25.2)	46 (43.0)	22 (20.6)	
Surgical NEC						
No	880	47 (5.3)	171 (19.4)	371 (42.2)	291 (33.1)	.82
Yes	38	2 (5.3)	9 (23.7)	12 (31.6)	15 (39.5)	
BPD						
No	602	29 (4.8)	103 (17.1)	251 (41.7)	219 (36.4)	<.001
Yes	296	20 (6.8)	73 (24.7)	127 (42.9)	76 (25.7)	

Abbreviations: BPD, bronchopulmonary dysplasia; IVH, intraventricular hemorrhage; NEC, necrotizing enterocolitis; ROP, retinopathy of prematurity.

^a^ Total number with the Apgar score will differ given missing values for individual covariables.

^b^ Data are given as percentages of rows separated for the Apgar score.

^c^ *P* values were computed after adjusting for country using ordered logistical regression models.

### Apgar Score and Neurodevelopmental Outcomes

**Figure 2.**
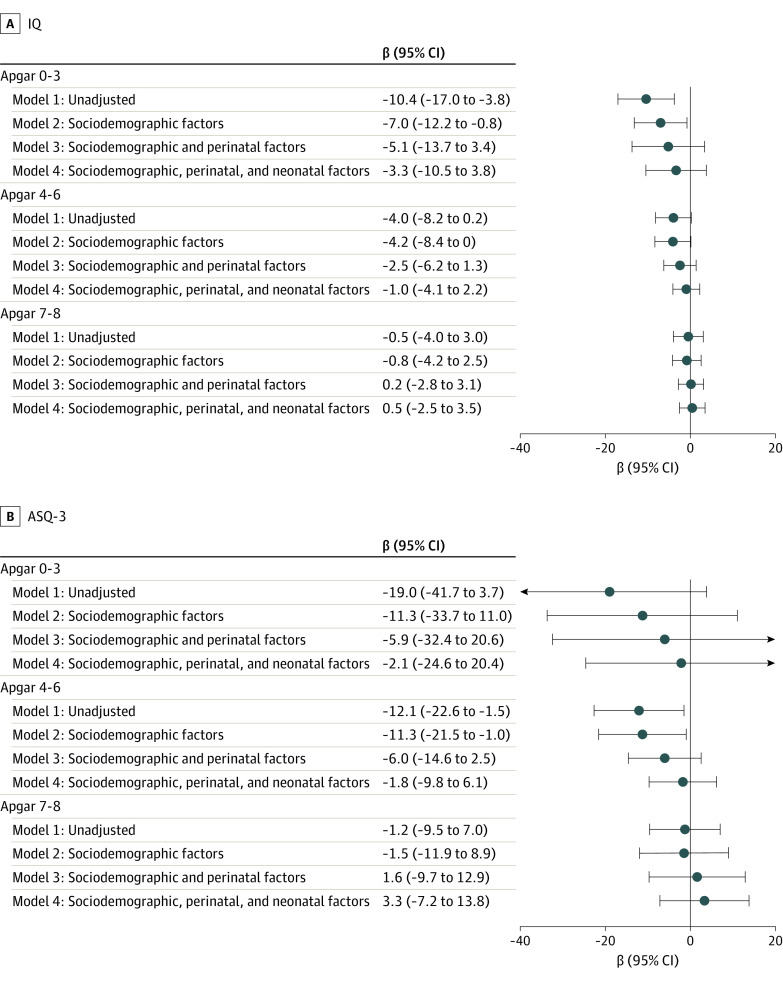
Association of Apgar Score Group and Cognitive Outcome The association of the Apgar score at 5 minutes of life and A, IQ as determined by Wechsler Preschool and Primary Scale of Intelligence–Revised, Third, or Fourth Edition and B, caregiver-reported Ages and Stages Questionnaire, third edition (ASQ-3) results was investigated using multinomial logistic regression with inverse probability weighting and multiple imputation. Data were analyzed without adjustments except for country as a fixed effect, with additional adjustment on sociodemographic factors, plus adjustment on perinatal factors and with additional adjustment on severe neonatal morbidities. The reference group is Apgar score 9 and 10.

Compared with children with an Apgar score of 9 to 10, those with scores of 0 to 3 had lower IQs (β = −10.4; 95% CI, −17.0 to −3.8) before risk adjustment and after risk adjustment for sociodemographic factors. Statistically significant differences were no longer present after adjustment on perinatal factors (β = −5.1; 95% CI, −13.7 to 3.4) or after adjusting for severe neonatal morbidities (β = −3.3; 95% CI, −10.5 to 3.8) (Figure 2). Similar patterns were observed in unadjusted and adjusted (β = −2.1; 95% CI, −24.6 to 20.4) analyses for the ASQ-3 score.

**Figure 3.**
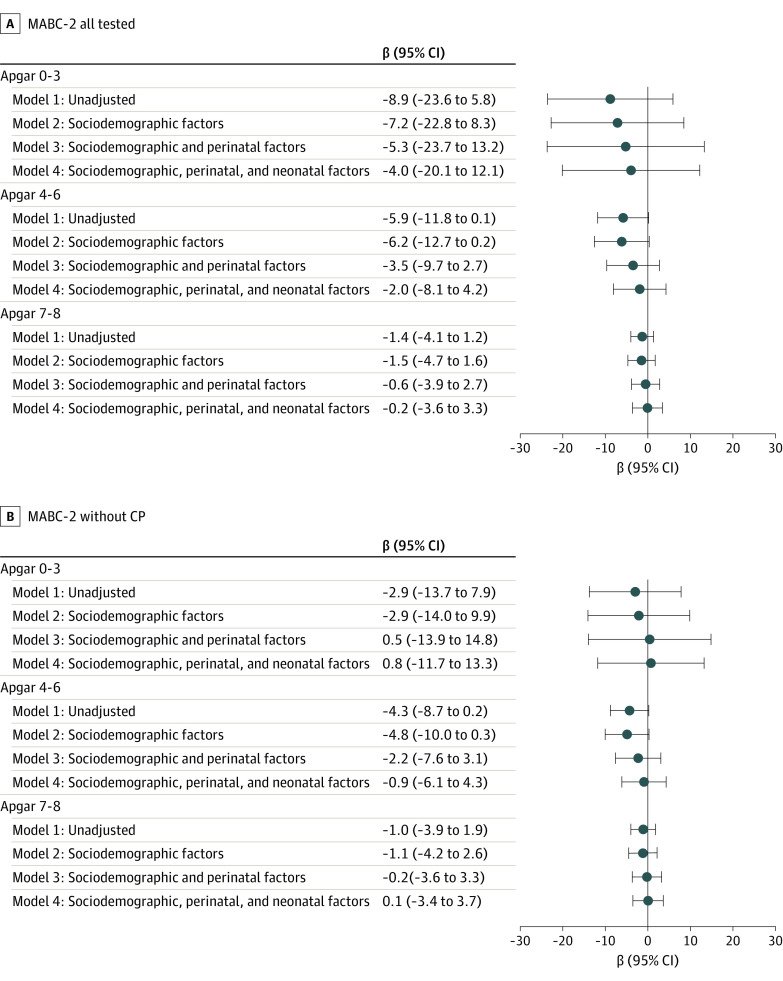
Association of Apgar Score Group and Motor Outcome The association of the Apgar score at 5 minutes of life and movement difficulties as determined by the Movement Assessment Battery for Children–Second Edition (MABC-2) and caregiver-reported questionnaires was investigated for A, global movement difficulties and B, movement difficulties not related to cerebral palsy using multinomial logistic regression with inverse probability weighting and multiple imputation. Data were analyzed without adjustments except for country as fixed effect, with additional adjustment on sociodemographic factors, plus adjustment on perinatal factors and with additional adjustment on severe neonatal morbidities. The reference group is Apgar score 9 and 10.

For motor function, there was no association before risk adjustment between the Apgar score and MABC-2 scores, even for the Apgar score of 0 to 3 (β = −8.9; 95% CI, −23.6 to 5.8) (Figure 3). Coefficients for lower Apgar score categories decreased and remained nonsignificant after risk adjustment for sociodemographic factors (Apgar score 0-3: β = −4.0; 95% CI, −20.1 to 12.1), further adjustment on baseline characteristics (Apgar score 0-3: β = −2.0; 95% CI, −8.1 to 4.2), and additional adjustment for severe neonatal morbidities (Apgar score 0-3: β = −0.2; 95% CI, −3.6 to 3.3) (Figure 3). Considering only children without CP, coefficients were lower than in the full sample and remained insignificant (Apgar score 0-3: β = 0.8; 95% CI, −11.7 to 13.3; Apgar score 4-6: β = −0.9; 95% CI, −6.1 to 4.3; Apgar score 7-8: β = 0.1; 95% CI, −3.4 to 3.7) (Figure 3). Results were similar when IPW and multiple imputation were removed, although the comparison of Apgar scores of 0 to 3 and 9 to 10 (β = −9.7; 95% CI, −18.9 to −0.5) for the MABC-2 score in the full population reached significance when considering all cases without multiple imputation (eFigures 1-4 in Supplement 1). Sensitivity analyses without multiple imputation for missing Apgar scores, on complete cases with IPW (eTables 2 and 3 in Supplement 1), and after exclusion of Belgium and Sweden, which differed in Apgar scoring from the other countries (eTables 2 and 3 in Supplement 1) did not change overall results from Figure 2 and Figure 3.

## Discussion

Despite improvements in the care of infants born EPT and increasing survival rates in this population, the risk for long-term neurodevelopmental impairment remains high, with a tremendous disease burden.^20,33,34,35,36,37,38,39^ Therefore, it is essential to identify infants at risk early in their life course to enable optimal decisions about care and early intervention. However, as this cohort study found, low Apgar scores at 5 minutes of life were not associated with impaired cognitive or motor outcomes at age 5 years, in contrast with results in infants born at term.^4,6,7,8,10,11^ Divergent outcomes from our study and those of infants born at term are in line with previous results on the association of low Apgar scores and risk of mortality, which found weaker associations at lower gestational ages.^5,12,13^ Our results complement previous studies on the prognostic value of the Apgar score in children born EPT, which support its utility for estimating survival but not longer-term neurodevelopmental outcomes.

Several hypotheses can be put forward for the unsuitability of the Apgar score as a prognostic marker associated with the longer-term outcome in infants born EPT in contrast to infants born at term. First, it was developed for infants born at term and those born EPT have generally reduced viability signs and poorer responses toward resuscitation after birth that are reflected by lower Apgar score values with decreasing gestational age.^5,12,13^ These facts cannot be naturally equated with postnatal fetal depression or interpreted to indicate insufficient resuscitation measures. Second, the Apgar score’s estimation accuracy for the outcome of infants born preterm may be hampered by intraobserver and interobserver variability, as well as differences in attitudes toward active resuscitation and in therapeutic measures in the delivery room between different centers and regions.^40,41,42,43,44^ Third, the risk of mortality is associated to a greater extent with the clinical condition after birth given that 50% of deaths among infants born EPT occur within the first 10 days of life.^45^ There were 851 deaths during the neonatal hospitalization and 17 deaths after discharge, and mortality selection may therefore contribute to a lack of association between the Apgar score and longer-term outcomes in this population and may have impacted our results. Furthermore, morbidities appearing later during the longitudinal course of neonatal intensive care unit therapy are affected by multiple and repeated exposures and therapies, including complications like late onset sepsis or frequent hypoxemic episodes.^46,47,48,49^ During this time, the largest increase in brain volume and expansion of the gray matter occurs, and vulnerability seems to differ across developmental stages that could moderate or override risk factors at birth. In any case, the discordance in the association of Apgar score with outcomes between children by gestational age at birth deserves further mechanistic explanation in future studies. Lastly, socioeconomic factors during the first 5 years of life are associated with a large change in neurodevelopmental outcomes, as detailed previously in our cohort,^23,29,50^ and may overshadow outcomes associated with perinatal risk factors.

The Apgar score distribution in our cohort was comparable to those in other large cohorts of EPT births.^12,13^ Our data are congruent with results from the International Neonatal Consortium analysis, in which no increased risk for IVH or CPVL was observed in association with low Apgar scores that constitute established associations with impairments in neurodevelopmental outcome.^12^ Of note, low Apgar scores have been associated with CP, as also found in a 2023 study from our cohort.^23,51,52,53,54^ In line with these findings, estimated coefficients were lower when children with CP were removed from our study. We also found significance for motor function when comparing groups with Apgar scores of 0 to 3 with those with scores of 9 to 10 in sensitivity analyses without IPW, which could reflect this association. Findings for IQ were robust in sensitivity analyses. Nonetheless, some estimates had wide CIs because of smaller numbers of children born with very low Apgar scores.

Our results stand in contrast to the documented association of low Apgar scores with neurodevelopmental outcomes in infants born at term.^4,6,7,8,9,10^ However, the most recent multicenter studies and meta-analyses^55,56^ substantiate the concerns of opponents of using low Apgar score values to estimate neurodevelopmental disability given that even an Apgar score of 0 at 10 minutes of life was a poor estimator, with at least half of surviving children having no disability or a mild disability. Therefore, the American Academy of Pediatrics (AAP) policy statement does not recommend the application of the Apgar score for resuscitation initiation and International Liaison Committee on Resuscitation (ILCOR) guidelines withdrew the suitability of the Apgar score as criterion to discontinue resuscitation measures in infants who are postnatally depressed.^2,57^ Furthermore, it is common research practice to consider the combined outcome of mortality and neurodevelopmental impairment. Our data indicate that it is important to separately consider the probability to survive and the chance for a life without severe impairment.

### Strengths and Limitations

The main strengths of our study are the longer-term follow-up evaluation that may provide better evidence for cognitive and motor outcomes, prospective approach based on predefined parameters and outcome definitions, and execution within 19 geographically and organizationally diverse regions across 11 European countries that may allow the broad application of results to a wide range of clinical care settings. The selected statistical approach with risk adjustment for severe neonatal morbidities associated with the neurodevelopmental outcome may provide an indication that the Apgar score did not give additional information on outcomes beyond its association with neonatal morbidities. Furthermore, we used IPW and multiple imputation to adjust for loss to follow-up and missing data, including missing Apgar scores.^32^

Thorough risk adjustments for variations in sociodemographic factors, baseline demographic parameters, perinatal characteristics, and severe neonatal morbidities made it possible to account for known factors associated with neurodevelopmental outcomes, and thus an association of Apgar score with the outcome is unlikely.^58^ We constructed models sequentially, starting with socioeconomic factors that may be associated with risk at birth and neurodevelopment at age 5 years but that would not be associated with Apgar score, perinatal risks present at birth, and a final model including neonatal morbidities, introduced separately given that these may act as mediators rather than confounders. This final model needs to be interpreted taking into consideration that neonatal morbidities are themselves associated with the Apgar score. This sequential approach allowed us to illustrate congruent results adjusting for perinatal as well as neonatal factors.

This study also has several limitations. Although we integrated many known risk factors, the number of documented items in the study was limited and we could not integrate information on race or ethnicity beyond foreign maternal birth or on the family context aside from maternal living situation and education and parental employment status.^21^ The low percentage of infants with Apgar scores of 0 to 3 and the restricted sample size compared with studies on preterm mortality constitute further restrictions of our analyses, but larger-scale clinical follow-up would not have been feasible.^5,12,13^ Additionally, there may have been varying attitudes of neonatal intensive care unit teams toward active care at less than 24 weeks’ gestation. Furthermore, we could not ascertain how sociodemographic status may have been associated with family support measures and the reluctance of parents to participate due to severe limitations of their infants and variations in follow-up service use at age 5 years in different regions.^29,59^ Further selection bias may have been introduced by the varying follow-up rates by country. Additionally, umbilical cord blood gas parameters were not available from all participating centers, prohibiting their inclusion in analyses.^60^

## Conclusions

While the Apgar score may be associated with in-hospital mortality and valuable for clinical risk assessment and counseling parents regarding survival, this cohort study found a disparity in its value for longer-term outcomes and may close a gap in knowledge on the longer-term outcomes associated with low Apgar scores in this population. Our results provide support for current AAP and ILCOR recommendations to not use the Apgar score for treatment decisions^2,57^ and may contribute to eliminating previous uncertainty on this topic.^61,62,63^ Future research should investigate whether initiatives to improve the utility of the Apgar score by the inclusion of further objective parameters, including ventilatory and cardiovascular interventions, can improve its predictive value.^64,65^ There remains an urgent need to establish precise prognostic parameters that support the clinician in the early identification of infants at risk for neurodevelopmental impairment to initiate therapeutic intervention programs that are known to improve the long-term outcome.

## Notes

Supplement 1. **eFigure 1.** Results for Wechsler Preschool and Primary Scale of Intelligence and Ages and Stages Questionnaire, Third Edition Without Multiple Imputation **eFigure 2.** Results for Wechsler Preschool and Primary Scale of Intelligence and Ages and Stages Questionnaire, Third Edition Without Inverse Probability Weighting and Multiple Imputation **eFigure 3.** Results for Global and Non–Cerebral Palsy Movement Difficulty Without Multiple Imputation **eFigure 4.** Results for Global and Non–Cerebral Palsy Movement Difficulty Without Inverse Probability Weighting and Multiple Imputation **eTable 1.** Description of 996 Children With Follow-Up at Age 5 y **eTable 2.** Sensitivity Analyses of Model 3: Adjustments for Country and Sociodemographic and Perinatal Factors **eTable 3.** Sensitivity Analyses of Model 4: Adjustments for Country and Sociodemographic and Perinatal Factors

Supplement 2. **Nonauthor Collaborators.** EPICE-SHIPS Research Group

Supplement 3.
Data Sharing Statement
