# Supplementary material for: Apgar Score and Neurodevelopmental Outcomes at Age 5 Years in Infants Born Extremely Preterm
Source: JAMA Netw Open. 2023 Sep 6;6(9):e2332413. doi: 10.1001/jamanetworkopen.2023.32413 (PMC10483322; doi:10.1001/jamanetworkopen.2023.32413)
Supplement: Supplement 1. — eFigure 1. Results for Wechsler Preschool and Primary Scale of Intelligence and Ages and Stages Questionnaire, Third Edition Without Multiple Imputation eFigure 2. Results for Wechsler Preschool and Primary Scale of Intelligence and Ages and Stages Questionnaire, Third Edition Without Inverse Probability Weighting and Multiple Imputation eFigure 3. Results for Global and Non–Cerebral Palsy Movement Difficulty Without Multiple Imputation eFigure 4. Results for Global and Non–Cerebral Palsy Movement Difficulty Without Inverse Probability Weighting and Multiple Imputation eTable 1. Description of 996 Children With Follow-Up at Age 5 y eTable 2. Sensitivity Analyses of Model 3: Adjustments for Country and Sociodemographic and Perinatal Factors eTable 3. Sensitivity Analyses of Model 4: Adjustments for Country and Sociodemographic and Perinatal Factors [file jamanetwopen-e2332413-s001.pdf]

Supplemental Online Content

Ehrhardt H, Aubert AM, Ådén U, et al; for the EPICE-SHIPS Research Group. Apgar score and neurodevelopmental outcomes at age 5 years in infants born extremely preterm. *JAMA Netw Open*. 2023;6(9):e2332413. doi:10.1001/jamanetworkopen.2023.32413

eFigure 1. Results for Wechsler Preschool and Primary Scale of Intelligence and Ages and Stages Questionnaire, Third Edition Without Multiple Imputation

eFigure 2. Results for Wechsler Preschool and Primary Scale of Intelligence and Ages and Stages Questionnaire, Third Edition Without Inverse Probability Weighting and Multiple Imputation

eFigure 3. Results for Global and Non–Cerebral Palsy Movement Difficulty Without Multiple Imputation

eFigure 4. Results for Global and Non–Cerebral Palsy Movement Difficulty Without Inverse Probability Weighting and Multiple Imputation

eTable 1. Description of 996 Children With Follow-Up at Age 5 y

eTable 2. Sensitivity Analyses of Model 3: Adjustments for Country and Sociodemographic and Perinatal Factors

eTable 3. Sensitivity Analyses of Model 4: Adjustments for Country and Sociodemographic and Perinatal Factors

This supplemental material has been provided by the authors to give readers additional information about their work.

© 2023 Ehrhardt H et al. *JAMA Network Open.*

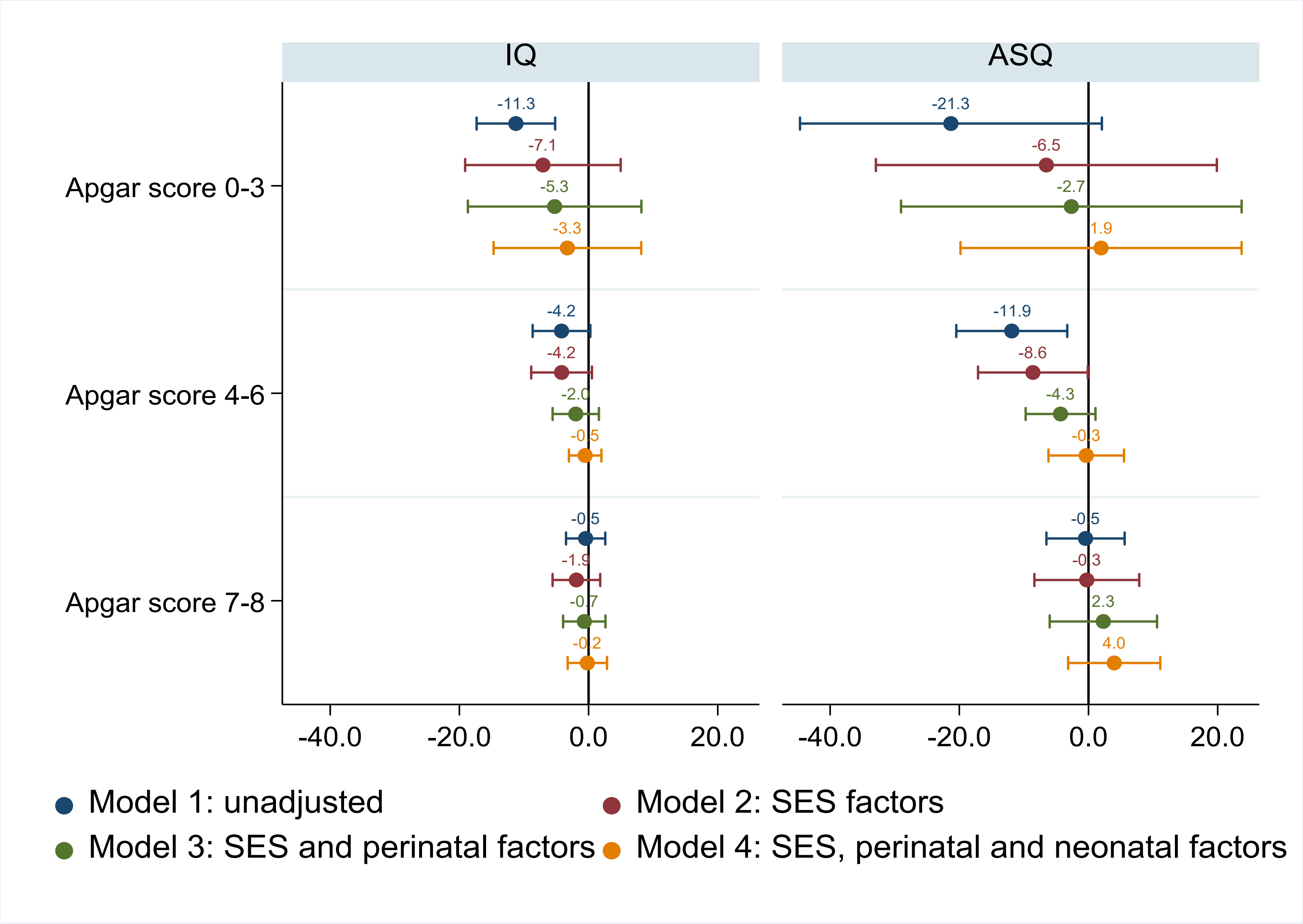

© 2023 Ehrhardt H et al. *JAMA Network Open.*

eFigure 1

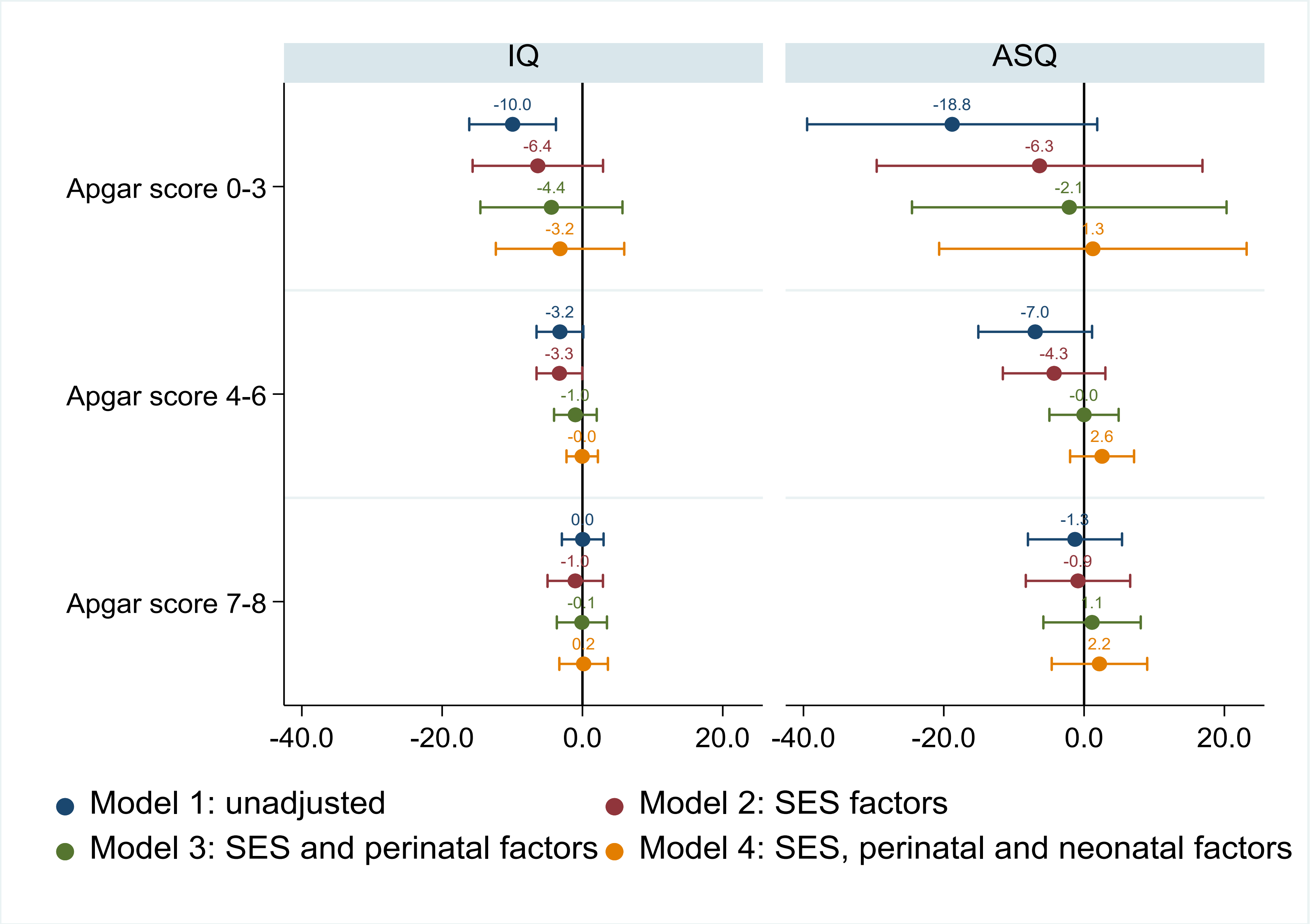

© 2023 Ehrhardt H et al. *JAMA Network Open.*

eFigure 2

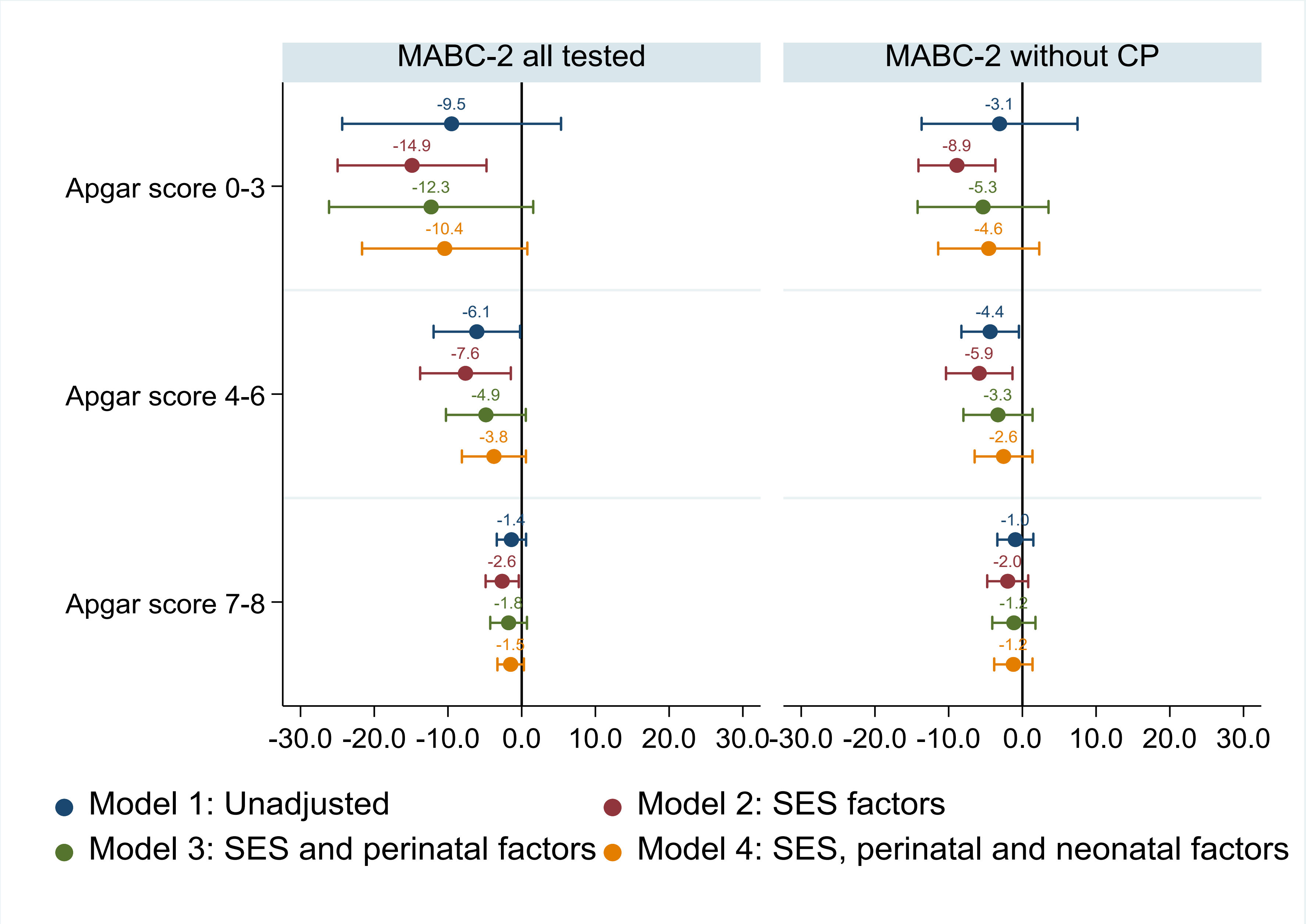

© 2023 Ehrhardt H et al. *JAMA Network Open.*

eFigure 3

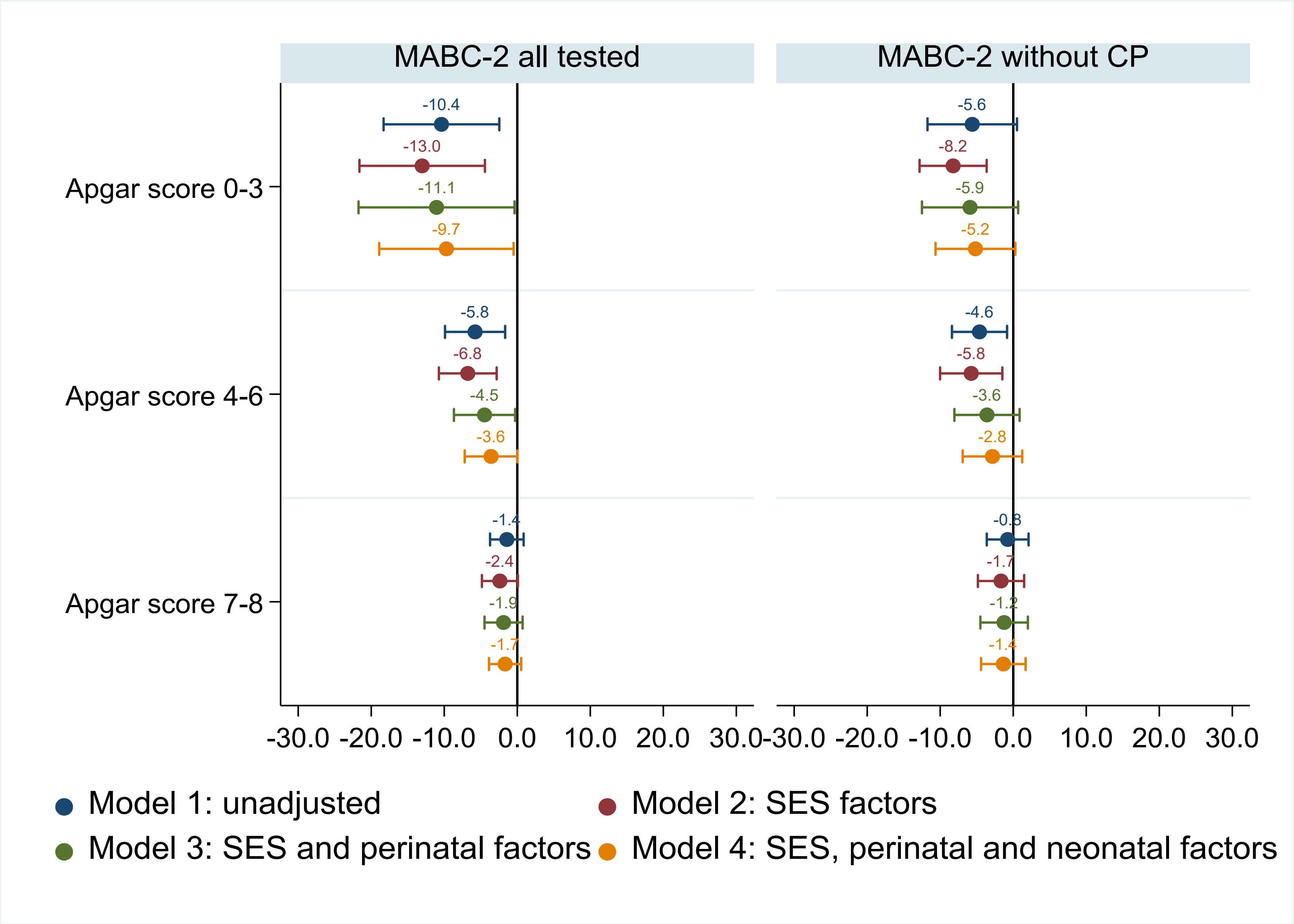

© 2023 Ehrhardt H et al. *JAMA Network Open.*

eFigure 4

eFigure Legends

eFigure 1. **Recalculation of association of Apgar score and mental outcome without multiple imputation.** The association of the Apgar score at 5 minutes of life and IQ determined by WPPSI (left panel) and caregiver reported ASQ3 questionnaire results (right panel) from Figure 2 were executed without multiple imputation. Data were analyzed without adjustments except for country as fixed effect (presented in blue color), with additional adjustment on sociodemographic factors (presented in red color), plus adjustment on perinatal factors (presented in green color) and with additional adjustment on severe acute neonatal morbidities (presented in orange color).

eFigure 2. **Recalculation of association of Apgar score and mental outcome without IPW and multiple imputation.** Results from eFigure 1 were executed without inverse probability weighting (IPW) and multiple imputation.

eFigure 3. **Recalculation of association of Apgar score and motor outcome without multiple imputation.** The association of the Apgar score at 5 minutes of life and global motor dysfunction (MD) including cerebral palsy determined by MABC-2 (left panel) and non-CP MD (right panel) from Figure 3 were executed without multiple imputation. Data were analyzed without adjustments except for country as fixed effect (presented in blue color), with additional adjustment on sociodemographic factors (presented in red color), plus adjustment on perinatal factors (presented in green color) and with additional adjustment on severe acute neonatal morbidities (presented in orange color).

eFigure 4**. Recalculation of association of Apgar score and motor outcome without IPW and multiple imputation.** Results from eFigure 3 were executed without inverse probability weighting (IPW) and multiple imputation.

© 2023 Ehrhardt H et al. *JAMA Network Open.*

eTables

eTable 1 Description of the sample of children with follow-up at 5 years of age (N=996)

Characteristics	N	%
Maternal age		
<25 years	115	11.6
25-34 years	593	59.8
n35 years	284	28.6
Missing	4	(0.4)
Living situation		
Single/Other	134	14.3
Married/Cohabiting	804	85.7
Missing	58	(5.8)
Education		
Low education ISCED	172	18.0
Intermediate education	426	44.6
High education ISCED	358	37.4
Missing	40	(4.0)
Parents employed		
Yes	806	86.7
No, 1 or both	124	13.3
Missing	66	(6.6)
Parity		
Primiparous	588	59.8
Multiparous	396	40.2
Missing	12	(1.2)
Country of birth		
Country	769	77.4
Other European	65	6.5
non-European	159	16.0
Missing	3	(0.3)
Gestational age		
24	132	13.3
25	185	18.6
26	282	28.3
27	397	39.9
Missing	0	(0.0)
SGA		
<3	154	15.5
3-9	81	8.1
≥ 10	761	76.4
Missing	0	(0.0)
Sex		
Male	518	52.0
Female	478	48.0
Missing	0	(0.0)
Type pregnancy		
Singleton	718	72.1
Multiple	278	27.9
Missing	0	(0.0)
PPROM		
No	727	73.9

© 2023 Ehrhardt H et al. *JAMA Network Open.*

Yes	257	26.1
Missing	12	(1.2)
ANS		
No	111	11.2
Yes	878	88.8
Missing	7	(0.7)
Any congential anomaly		
No	912	91.6
Yes	84	8.4
Missing	0	(0.0)
IVH or cPVL		
No	853	86.3
Yes	135	13.7
Missing	8	(0.8)
ROP		
No	861	87.7
Yes	121	12.3
Missing	14	(1.4)
Surgical NEC		
No	954	95.8
Yes	42	4.2
Missing	0	(0.0)
BPD		
No	323	32.4
Yes	24	2.4
Missing	0	(0.0)
Country		
Belgium	70	7.0
Denmark	52	5.2
Estonia	38	3.8
France	167	16.8
Germany	78	7.8
Italy	173	17.4
Netherlands	75	7.5
Poland	52	5.2
Portugal	111	11.1
United Kingdom	138	13.9
Sweden	42	4.2

© 2023 Ehrhardt H et al. *JAMA Network Open.*

eTable 2. Sensitivity analyses of final models – Model 3 (adjustments for country, on sociodemographic and perinatal factors)

Model	Final model IM and IPW	Without MI for Apgar with IPW	Complete Cases With IPW	Complete Cases without IPW	Final model without BE or SE
IQ	892	822	729	729	797
1-3	-5.1 (-13.7-3.4)	-5.2 (-13.9-3.4)	-5.3 (-18.7-8.2)	-4.4 (-14.6-5.7)	-4.4 (-14.9-6.2)
4-6	-2.5 (-6.2-1.3)	-2.7 (-6.5-1.1)	-2 (-5.6-1.6)	-1.0 (-4.1-2.0)	-2.7 (-6.9-1.5)
7-8	0.2 (-2.8-3.1)	0.2 (-2.2-2.6)	-0.7 (-3.9-2.6)	-0.1 (-3.7-3.5)	-0.2 (-3.6-3.1)
ASQ	764	702	647	647	663
1-3	-5.9 (-32.4-20.6)	-7.5 (-35.5-20.5)	-2.7 (-29-23.7)	-2.1 (-24.5-20.3)	0.3 (-34.8-35.4)
4-6	-6.0 (-14.6-2.5)	-6.0 (-12.6-0.6)	-4.3 (-9.7-1.1)	0.0 (-4.9-4.9)	-7.9 (-27.9-2.1)
7-8	1.6 (-9.7-12.9)	2.0 (-7.3-11.4)	2.3 (-6-10.6)	1.1 (-5.8-8.1)	0.5 (-13.5-14.5)
MABC-2 all tested	818	755	677	677	746
1-3	-5.3 (-23.7-13.2)	-5.8 (-25.7-14.1)	-12.3 (-26.1-1.6)	-11.1 (-21.7--0.4)	-3.6 (-28-20.9)
4-6	-3.5 (-9.7-2.7)	-4.0 (-10.4-2.4)	-4.9 (-10.3-0.6)	-4.5 (-8.7--0.3)	-2.7 (-9.4-3.9)
7-8	-0.6 (-3.9-2.7)	-0.7 (-3.8-2.3)	-1.8 (-4.3-0.7)	-1.9 (-4.5-0.7)	0 (-3.5-3.5)
MABC-2 without CP	771	713	638	638	705
1-3	0.5 (-13.9-14.8)	0.4 (-15-15.8)	-5.3 (-14.2-3.5)	-5.9 (-12.5-0.7)	1.6 (-17.1-20.3)
4-6	-2.2 (-7.6-3.1)	-2.6 (-8-2.7)	-3.3 (-8-1.4)	-3.6 (-8.1-0.9)	-1.1 (-6.2-4.1)
7-8	-0.2 (-3.6-3.3)	-0.2 (-3.5-3.1)	-1.2 (-4.1-1.8)	-1.2 (-4.5-2)	0.7 (-2.9-4.2)

Analyses of model 3 from Figure 2 and 3 were rerun for IQ, MABC-2 and MABC-2 without CP without multiple imputation (IM) for the Apgar score and for complete cases with and without inverse probability weighting (IPW) and excluding Belgium (BE) and Sweden (SE).

© 2023 Ehrhardt H et al. *JAMA Network Open.*

eTable 3. Sensitivity analyses of final models – Model 4 (adjustments for country, on sociodemographic, perinatal and neonatal factors)

Model	Final model IM and IPW	Without MI for Apgar with IPW	Complete Cases With IPW	Complete Cases without IPW	Final model without BE and SE
IQ	892	822	707	707	797
1-3	-3.3 (-10.5-3.8)	-3.7 (-26.7-19.3)	-3.3 (-14.7-8.2)	-3.2 (-12.3-6)	-3.0 (-12.1-6.1)
4-6	-1.0 (-4.1-2.2)	-1.6 (-7.2-3.9)	-0.5 (-3-2)	0.0 (-2.3-2.2)	-1.4 (-5.1-2.3)
7-8	0.5 (-2.5-3.5)	3.9 (-4.5-12.3)	-0.2 (-3.2-2.9)	0.2 (-3.3-3.6)	-0.1 (-3.4-3.3)
ASQ	764	702	660	660	663
1-3	-2.1 (-24.6-20.4)	-3.7 (-26.7-19.3)	1.9 (-19.8-23.7)	1.3 (-20.7-23.2)	3.7 (-24.3-31.8)
4-6	-1.8 (-9.8-6.1)	-1.6 (-7.2-3.9)	-0.3 (-6.2-5.5)	2.6 (-2-7.1)	-3.4 (-13.1-6.2)
7-8	3.3 (-7.2-13.8)	3.9 (-4.5-12.3)	4 (-3.2-11.1)	2.2 (-4.6-9)	1.9 (-11.1-14.9)
MABC-2 all tested	818	755	655	655	746
1-3	-4.0 (-20.1-12.1)	-4.8 (-22.3-12.7)	-10.4 (-21.7-0.8)	-9.7 (-18.9--0.5)	-2.7 (-24-18.5)
4-6	-2.0 (-8.1-4.2)	-2.4 (-8.8-4)	-3.8 (-8.1-0.6)	-3.6 (-7.2-0)	-1.2 (-8.1-5.7)
7-8	-0.2 (-3.6-3.3)	-0.2 (-3.5-3)	-1.5 (-3.3-0.3)	-1.7 (-3.9-0.5)	0.4 (-3.6-4.3)
MABC-2 without CP	771	713	617	617	705
1-3	0.8 (-11.7-13.3)	0.5 (-13-13.9)	-4.6 (-11.4-2.3)	-5.2 (-10.6-0.3)	1.9 (-13.6-17.4)
4-6	-0.9 (-6.1-4.3)	-1.2 (-6.4-4)	-2.6 (-6.5-1.4)	-2.8 (-6.9-1.2)	0.3 (-5.1-5.6)
7-8	0.1 (-3.4-3.7)	0.1 (-3.3-3.5)	-1.2 (-3.8-1.4)	-1.4 (-4.4-1.7)	0.9 (-2.9-4.7)

Analyses of model 4 from Figure 2 and 3 were rerun for IQ, MABC-2 and MABC-2 without CP without multiple imputation (IM) for the Apgar score and for complete cases with and without inverse probability weighting (IPW) and excluding Belgium (BE) and Sweden (SE).

© 2023 Ehrhardt H et al. *JAMA Network Open.*
