# Supplementary material for: Apgar Score and Neurodevelopmental Outcomes at Age 5 Years in Infants Born Extremely Preterm
Source: JAMA Netw Open. 2023 Sep 6;6(9):e2332413. doi: 10.1001/jamanetworkopen.2023.32413 (PMC10483322; doi:10.1001/jamanetworkopen.2023.32413)
Supplement: Supplement 2. — Nonauthor Collaborators. EPICE-SHIPS Research Group [file jamanetwopen-e2332413-s002.pdf]

*Group Name(s): EPICE-SHIPS Research Group							
***First Name and Middle Initial(s)**	***Last Name**	***Suffix (eg, Jr, III)**	Academic Degrees	Institution	Location (city, state/province, country)	Role or Contribution, eg, chair, principal investigator	Group (if more than 1 Group listed in the byline) and/or Subgroup (eg, Steering Committee)
Jo	Lebeer		MD	Department of Family Medicine & Population Health (FAMPOP), Disability Studies, Faculty of Medicine & Health Sciences, University of Antwerp	Antwerp, Belgium	principle investigator	
Patrick	van Reempts		PhD	Laboratory of Experimental Medicine and Pediatrics, Division of Neonatology and Study Centre for Perinatal Epidemiology Flanders, University of Antwerp	Brussels, Belgium	co-investigator	
Els	Bruneel		MD	Centre for Developmental Disabilities, Neonatal Intensive Care, Oost Limburg Hospital	Genk, Belgium	co-investigator	
Eva	Cloet		PhD	Vrije Universiteit Brussel Faculteit Geneeskunde en Farmacie, Paediatric Neurology, Universitair Ziekenhuis Brussels	Brussels, Belgium	co-investigator	
Ann	Oostra			Centre for Developmental Disabilities, Ghent University Hospital	Ghent, Belgium	co-investigator	
Els	Ortibus			Centre for Developmental Disabilities, Leuven University Hospital and Department of Neuropediatrics, University of Leuven	Leuven, Belgium	co-investigator	

***First Name and Middle Initial(s)**	***Last Name**	***Suffix (eg, Jr, III)**	Academic Degrees	Institution	Location (city, state/province, country)	Role or Contribution, eg, chair, principal investigator	Group (if more than 1 Group listed in the byline) and/or Subgroup (eg, Steering Committee)
Iemke	Sarrechia		PhD	Centre for Developmental Disabilities, Antwerp University Hospital and Department of Primary & Interdisciplinary Care, Disability Studies, Faculty of Medicine, University of Antwerp	Antwerp, Belgium	co-investigator	
Klaus	Boerch		MD	Department of Paediatrics, Hvidovre Hospital, Copenhagen University Hospital	Hvidovre, Denmark	co-investigator	
Lene	Huusom		MD	Department of Gynaecology and Obstetrics, Hvidovre University Hospital	Hvidovre, Denmark	co-investigator	
Pernille	Pedersen		MD	Department of Neonatology, Hvidovre Hospital	Hvidovre, Denmark	principle investigator	
Asbjørn	Hasselager		MD	Department of Paediatrics, Hvidovre University Hospital	Copenhagen, Denmark	co-investigator	
Liis	Toome		MD	Tallinn Children’s Hospital and University of Tartu	Tartu, Estonia	associated partner	
Mairi	Männamaa		PhD	Department of Paediatrics, Institute of Clinical Medicine, University of Tartu	Tartu, Estonia	co-investigator	
Pierre-Yves	Ancel		PhD	Université Paris Cité, Inserm, INRAE, Centre for Research in Epidemiology and StatisticS (CRESS), Obstetrical Perinatal and Pediatric Epidemiology Research Team, EPOPé	Paris, France	co-investigator	
Antoine	Burguet		MD	Division of Pediatrics 2, Hôpital du Bocage, INSERM CIE1, CHRU Dijon, Université de Dijon	Dijon, France	co-investigator	

***First Name and Middle Initial(s)**	***Last Name**	***Suffix (eg, Jr, III)**	Academic Degrees	Institution	Location (city, state/province, country)	Role or Contribution, eg, chair, principal investigator	Group (if more than 1 Group listed in the byline) and/or Subgroup (eg, Steering Committee)
Pierre	Jarreau		MD	Université Paris Descartes and Assistance Publique Hôpitaux de Paris, Hôpitaux Universitaire Paris Centre Site Cochin, DHU Risks in Pregnancy, Service de Médecine et Réanimation néonatales de Port-Royal	Paris, France	co-investigator	
Véronique	Pierrat		MD	Université Paris Cité, Inserm, INRAE, Centre for Research in Epidemiology and StatisticS (CRESS), Obstetrical Perinatal and Pediatric Epidemiology Research Team, EPOPé and Department of Neonatology, CHI Créteil	Paris, France; Créteil, France	co-investigator	
Patrick	Truffert		MD	Department of Neonatology, Jeanne de Flandre Hospital, Lille CHRU	Lille, France	co-investigator	
Björn	Misselwitz		MD	Institute of Quality Assurance Hesse	Eschborn, Germany	co-investigator	
Stephan	Schmidt		MD	Department of Obstetrics, University Hospital, Philipps University	Marburg, Germany	co-investigator	
Lena	Wohlers		MD	Children’s Hospital, University Hospital, Philipps University Marburg	Marburg, Germany	co-investigator	
Maria	Cuttini		MD	Clinical Care and Management Innovation Research Area, Bambino Gesù Children’s Hospital, IRCCS	Rome, Italy	principle investigator	
Domenico	Di Lallo		MD	Lazio Regional Health Authority	Rome, Italy	co-investigator	

***First Name and Middle Initial(s)**	***Last Name**	***Suffix (eg, Jr, III)**	Academic Degrees	Institution	Location (city, state/province, country)	Role or Contribution, eg, chair, principal investigator	Group (if more than 1 Group listed in the byline) and/or Subgroup (eg, Steering Committee)
Gina	Ancora		MD	Neonatal Intensive Care Unit, Ospedale degli Infermi	Rimini, Italy	co-investigator	
Dante	Baronciani		MD	General Directorate for Health and Social Policies, Emilia Romagna Region, Bologna and Centre for Evaluation off Efficasy in Health Care-CeVAS	Modena, Italy	co-investigator	
Virgilio	Carnielli		PhD	Maternal and Child Health Institute, Marche University and Salesi Hospital	Ancona, Italy	co-investigator	
Ileana	Croci		MsC	Clinical Care and Management Innovation Research Area, Bambino Gesù Children's Hospital, IRCCS	Roma, Italy	co-investigator	
Giacomo	Faldella		MD	Neonatal Intensive Care Unit, University Hospital S. Orsola-Malpighi	Bologna, Italy	co-investigator	
Frederica	Ferrari		MD	Department of Pediatrics and Neonatology, Modena University Hospital	Modena, Italy	co-investigator	
Francesco	Franco		MsC	Regional Health Agency of Lazio	Rome, Italy	co-investigator	
Giancarlo	Gargano		MD	Arcispedale Santa Maria Nuova, IRCCS	Reggio Emilia, Italy	co-investigator	
Arno	van Heijst		MD	Department of Neonatology, Radboud University Medical Center	Nijmegen, the Netherlands	principle investigator	
Corine	Koopman-Esseboom		MD	Department of Neonatology, Wilhelmina Children's Hospital	Utrecht, the Netherlands	associated partner	

***First Name and Middle Initial(s)**	***Last Name**	***Suffix (eg, Jr, III)**	Academic Degrees	Institution	Location (city, state/province, country)	Role or Contribution, eg, chair, principal investigator	Group (if more than 1 Group listed in the byline) and/or Subgroup (eg, Steering Committee)
Janusz	Gadzinowski		MD	Department of Neonatology, Poznan University of Medical Sciences	Poznan, Poland	principle investigator	
Jan	Mazela		MD	Department of Neonatology and Neonatal Infectious Diseases, Poznan University of Medical Sciences	Poznan, Poland	co-investigator	
Alan	Montgomery		PhD	Department of Neonatology, Poznan University of Medical Sciences	Poznan, Poland	co-investigator	
Tomasz	Pikuła		MD	Department of Neonatology, Poznan University of Medical Sciences	Poznan, Poland	co-investigator	
Henrique	Barros		MD	EPIUnit-Institute of Public Health, University of Porto	Porto, Portugal	principle investigator	
Raquel	Costa		PhD	EPIUnit-Institute of Public Health, University of Porto	Porto, Portugal	co-investigator	
Luís	Graça		MD	University Hospital of Santa Maria, Faculty of Medicine, University of Lisbon	Lisbon, Portugal	co-investigator	
Maria	do Céu Machado		MD	University of Lisbon and Departamento de Pediatria do Hospital Santa Maria	Lisbon, Portugal	co-investigator	
Carina	Rodrigues		PhD	EPIUnit-Institute of Public Health, University of Porto	Porto, Portugal	co-investigator	
Teresa	Rodrigues		PhD	EPIUnit-Institute of Public Health, University of Porto	Porto, Portugal	co-investigator	

***First Name and Middle Initial(s)**	***Last Name**	***Suffix (eg, Jr, III)**	Academic Degrees	Institution	Location (city, state/province, country)	Role or Contribution, eg, chair, principal investigator	Group (if more than 1 Group listed in the byline) and/or Subgroup (eg, Steering Committee)
Anna-Karin	Edstedt Bonamy		PhD	Clinical Epidemiology Division, Department of Medicine Solna, Karolinska Institutet and Department of Women's and Children's Health, Karolinska Institutet	Stockholm, Sweden	co-investigator	
Mikael	Norman		MD	Department of Clinical Science and Department of Neonatal Medicine, Karolinska University Hospital	Stockholm, Sweden	co-investigator	
Elaine	Boyle		MD	Department of Health Sciences, University of Leicester	Leicester, United Kingdom	co-investigator	
Alan	Fenton		MD	Newcastle Neonatal Service, Newcastle University	Newcastle upon Tyne, United Kingdom	co-investigator	
Samantha	Johnson		PhD	Department of Health Sciences, University of Leicester	Leicester, United Kingdom	co-investigator	
Bradley	Manktelow		PhD	Department of Health Sciences, University of Leicester	Leicester, United Kingdom	co-investigator	
David	Milligan		MD	Faculty of Medical Science, Newcastle University	Newcastle upon Tyne, United Kingdom	associated partner	
Silke	Mader			European Foundation for the Care of Newborn Infants (EFCNI)	Munich, Germany	partner	
Nicole	Thiele			European Foundation for the Care of Newborn Infants (EFCNI)	Munich, Germany	partner	
Johanna	Walz		MD	European Foundation for the Care of Newborn Infants (EFCNI)	Munich, Germany	partner	
Stavros	Petrou		PhD	Nuffield Department of Primary Care Health Sciences, University of Oxford	Oxford, United Kingdom	principle investigator	

***First Name and Middle Initial(s)**	***Last Name**	***Suffix (eg, Jr, III)**	Academic Degrees	Institution	Location (city, state/province, country)	Role or Contribution, eg, chair, principal investigator	Group (if more than 1 Group listed in the byline) and/or Subgroup (eg, Steering Committee)
Mercedes	Bonet		PhD	Université Paris Cité, Inserm, INRAE, Centre for Research in Epidemiology and StatisticS (CRESS), Obstetrical Perinatal and Pediatric Epidemiology Research Team, EPOPé	Paris, France	co-investigator	
Camille	Bonnet		PhD	Université Paris Cité, Inserm, INRAE, Centre for Research in Epidemiology and StatisticS (CRESS), Obstetrical Perinatal and Pediatric Epidemiology Research Team, EPOPé	Paris, France	co-investigator	
Rym	El Raffei			Université Paris Cité, Inserm, INRAE, Centre for Research in Epidemiology and StatisticS (CRESS), Obstetrical Perinatal and Pediatric Epidemiology Research Team, EPOPé	Paris, France	project manager	
Aurélie	Piedvache		MsC	Université Paris Cité, Inserm, INRAE, Centre for Research in Epidemiology and StatisticS (CRESS), Obstetrical Perinatal and Pediatric Epidemiology Research Team, EPOPé and Department of Neonatology, CHI Créteil	Paris, France and Créteil, France	co-investigator	
Anna-Veera	Seppänen		PhD	Université Paris Cité, Inserm, INRAE, Centre for Research in Epidemiology and StatisticS (CRESS), Obstetrical Perinatal and Pediatric Epidemiology Research Team, EPOPé	Paris, France	project manager	
