# Supplementary material for: Apgar Score and Neurodevelopmental Outcomes at Age 5 Years in Infants Born Extremely Preterm
Source: JAMA Netw Open. 2023 Sep 6;6(9):e2332413. doi: 10.1001/jamanetworkopen.2023.32413 (PMC10483322; doi:10.1001/jamanetworkopen.2023.32413)
Supplement: Supplement 3. — Data Sharing Statement [file jamanetwopen-e2332413-s003.pdf]

Data Sharing Statement

Ehrhardt. Apgar Score and Neurodevelopmental Outcomes at Age 5 Years in Infants Born Extremely Preterm. *JAMA Netw Open*. Published online September 6, 2023. doi:10.1001/jamanetworkopen.2023.32413

Data
Data available: No

Additional Information
Explanation for why data not available: Access to data in the EPICE and SHIPS cohort is currently not possible for researchers who are not members of the consortium, but EPICE is part of a H2020 project (RECAP, https://recap-preterm.eu/) to develop a platform for data sharing. The corresponding author is available for more information.
